# Perceived organizational effectiveness, moral injury, and moral resilience among nurses during the COVID-19 pandemic: Secondary analysis

**DOI:** 10.1097/01.NUMA.0000834524.01865.cc

**Published:** 2022-07-01

**Authors:** Cynda Hylton Rushton, Katie E. Nelson, Inga Antonsdottir, Ginger C. Hanson, Danielle Boyce

**Affiliations:** At Johns Hopkins University in Baltimore, Md., **Cynda Hylton Rushton** is the Anne and George L. Bunting Professor of Clinical Ethics, Nursing and Medicine at the Berman Institute of Bioethics & School of Nursing; **Katie Nelson** is a PhD candidate; **Inga Antonsdottir** is the program coordinator for the COVID Dementia Caregivers Supplement Project, and a graduate research assistant/coordinator for the Memory and Aging Services Innovation (MASI) Center in the Department of Psychiatry & Behavioral Sciences; **Ginger C. Hanson** is an assistant professor, and **Danielle Boyce** is an assistant professor, neurology.

## Abstract

It's crucial to understand the perspective of nurses during the pandemic to determine actionable steps for moving forward. This analysis looks at nurses' perceptions of their organizations' effectiveness during the first surge of the COVID-19 pandemic and its impact on moral injury and moral resilience.

**Figure:**
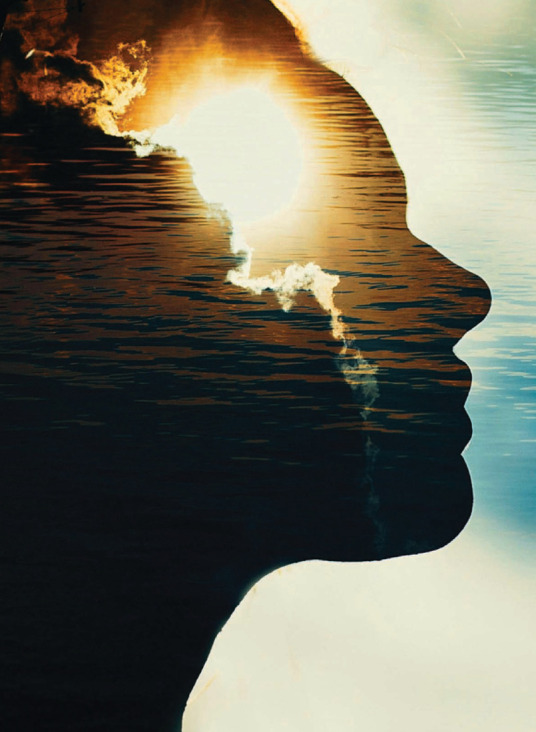
No caption available.

The COVID-19 pandemic has left many nurses in the US reconsidering or leaving the nursing profession due to questionable safety and organizational standards on top of pervasive burnout over the last 24 months.^1^-^3^ Those at the point of care are experiencing significant moral injury (MI)–defined as a profound threat or violation of one's moral foundation and conscience–which occurs in response to severe personal, collective, organizational, or leadership transgressions or betrayals.^4^,^5^ It can lead to erosion of one's moral identity and ignite feelings of guilt, shame, or unworthiness.^4^ Nurses providing care to patients with COVID-19 have faced undue moral burden, while concurrently navigating substantial resource shortages, taking on additional responsibilities, and in some cases, feeling left in the dark by their organizations and leaders.^4^,^6^-^8^ Breaches in organizational trust have eroded moral community, which can often escalate and/or perpetuate symptoms of MI.^9^,^10^ Moral resilience (MR), the capacity to preserve or restore integrity in response to moral adversity, has been posited as a protective resource to support nurses in meeting the unavoidable ethical challenges that accompany clinical care.^7^,^11^ Given nurses' critical role in providing patient care, their perspective is required to determine actionable steps and systematic processes for moving forward during an ongoing, ever-evolving pandemic. Therefore, the purpose of this secondary analysis was to understand the impact of nurses' perceptions of their organizations' effectiveness in relation to their MI and MR scores during the first surge (Alpha variant) of the COVID-19 pandemic.

## Methods

A survey was distributed from June through November of 2020 to elicit experiences of MR, MI, and organizational factors from a convenience sample of healthcare workers (HCWs) during the COVID-19 pandemic (N = 595). Those included in the sample were nurses, physicians, advanced practice providers, and other clinical staff over 18 years of age; any pediatric providers were excluded. Characteristics of the full dataset are described in a separate paper.^4^ For this analysis, data were limited to nurses from the original sample (N = 344). The Johns Hopkins Clinical Research Network, a network of academic and community-based medical centers in Maryland, Pennsylvania, and the District of Columbia, distributed the survey invitation and a web-based link to complete it. The Johns Hopkins Institutional Review Board deemed this study to be exempt; survey completion implied consent to participate.

### Measures

Organizational effectiveness (OE) was measured using 10 items developed by three subject-matter experts in medicine, bioethics, and nursing and confirmed via comprehensive literature review. Items were rated on a scale from 1 (not at all effective) to 5 (extremely effective). The OE score was calculated by summing responses to the 10 questions. Construct validity of the 10-item score was demonstrated using factor analysis, whereby all items loaded onto a single factor with factor loadings greater than .50, which explained 49.55% of the variability (alpha = .93). Items were dichotomized by the two highest and three lowest choices on the scale (0 = not at all/slightly/moderately effective, 1 = very/extremely effective), as in previous analysis.^4^

MR was measured using a shortened version of the *Rushton Moral Resilience Scale*.^12^ Through factor analysis, the scale was reduced to four items that loaded onto a single factor with factor loadings greater than .50, which explained 65.75% of the variability in the items (alpha = .74). Higher scores indicate greater MR.

Finally, MI was measured using the 10-item *Moral Injury Symptom Scale-Healthcare Professionals* (MISS-HP; alpha = .93).^13^ Higher scores indicated greater MI, and scores of 36 were considered the cutoff point for clinically significant MI.^13^

### Data analysis

We analyzed the data using statistical software and summarized descriptive statistics, including means, SD, and percentages, to characterize sample demographics. Means and SD are presented to show which facets of OE contributed to higher MI scores. We conducted two sets of analyses on the 10 OE items at the bivariate level (0 = not at all/slightly/moderately effective versus 1 = very/extremely effective). The first set of chi-square analyses examined the relationship between providing COVID-19 care and the 10 OE facets. Next, we conducted independent t-tests to examine whether there were significant differences in MR or MI depending on participants' responses to the OE items.

For the main analysis, a model building approach was implemented to reduce multicollinearity among variables. The bivariate analyses were conducted examining the relationship between one hypothesized antecedent with our dependent variable, MI. Variables that were significant in preliminary bivariate analyses were then added to a multiple regression model using the PROCESS utility.^14^ The PROCESS approach enabled us to also assess OE as a mediator of providing COVID-19 clinical care and MI score.

## Results

Characteristics of the nurse respondents (N = 344) are highlighted in Table 1. Most participants were involved in COVID-19 care (63.1%). The overall prevalence of clinically significant MI scores was 38.1%.

**Table 1:** Sample characteristics (N = 344)

Characteristic/Question	Answer	Mean (SD)
MI score		33.39 (13.77)
MR score		28.93 (5.02)
		**n (%)**
MI ≥ 36	No	190 (61.9)
	Yes	117 (38.1)
How many years have you worked in this profession?	Less than 3 years	34 (9.9)
	About 3-5 years	26 (7.6)
	About 5-10 years	64 (18.7)
	About 10-15 years	43 (12.5)
	About 15-20 years	31 (9)
	Longer than 20 years	145 (42.3)
What is your ethnicity?	Native American/Alaskan Native	0 (0)
	Asian/Pacific Islander	8 (2.7)
	Black	15 (5.1)
	White	246 (83.1)
	Multiple races	9 (3)
	Prefer not to answer	18 (6.1)
What is the highest level of education you've completed?	Associate degree	72 (21)
	Bachelor's degree	205 (59.8)
	Master's degree	64 (18.7)
	Doctorate degree	2 (0.6)
What is your spiritual/religious preference?	Buddhist	3 (0.9)
	Christian/Protestant	156 (45.3)
	Hindu	1 (0.3)
	Islam	1 (0.3)
	Roman Catholic	95 (27.6)
	Jewish	6 (1.7)
	Spiritual, not religious	42 (12.2)
	No religious preference	40 (11.6)
Religious/spiritual versus not religious/spiritual	Not religious/spiritual	40 (11.6)
	Religious/spiritual	304 (88.4)
What is your practice location?	ED	30 (8.8)
	Inpatient–CCU	63 (18.5)
	Inpatient–other	155 (45.5)
	OR	15 (4.4)
	Outpatient/ambulatory care	78 (22.9)
Are you involved in COVID-19 clinical care?	No	127 (36.9)
	Yes	217 (63.1)

### Highest facets of MI

To illuminate which facets of MI were highest among nurses during the COVID-19 pandemic, we present the means and standard deviations of each item in Table 2. The five top scoring facets of MI were: 1) reduced helpfulness of religious/spiritual faith, 2) feeling betrayed by other health professionals who were once trusted, 3) difficulty forgiving yourself for what's happened to you or others you've cared for, 4) guilt over failing to save someone from being seriously injured or dying, and 5) lack of trust in other health professional colleagues.

**Table 2:** Responses to individual moral injury scale items, nurses only (N = 344)

Scale items (corresponding response scales)	Mean (SD)
Compared to before you went through these experiences, has your religious/spiritual faith... (1 = Weakened a lot–10 = Strengthened a lot)∗	5.07 (2.48)
I feel betrayed by other health professionals I once trusted. (1 = Strongly disagree–10 = Strongly agree)	4.51 (3.24)
I've forgiven myself for what's happened to me or others I've cared for.∗ (1 = Strongly disagree–10 = Strongly agree)	4.42 (2.63)
I feel guilt over failing to save someone from being seriously injured or dying. (1 = Strongly disagree–10 = Strongly agree)	3.62 (2.97)
Most people with whom I work as a health professional are trustworthy.∗ (1 = Strongly disagree–10 = Strongly agree)	3.23 (2.33)
I'm troubled by having acted in ways that violated my own morals or values. (1 = Strongly disagree–10 = Strongly agree)	2.98 (2.63)
I feel ashamed about what I've done or not done when providing care to my patients. (1 = Strongly disagree–10 = Strongly agree)	2.93 (2.61)
I have a good sense of what makes my life meaningful as a health professional.∗ (1 = Absolutely untrue–10 = Absolutely true)	2.72 (2.15)
All in all, I'm inclined to feel that I'm a failure in my work as a health professional. (1 = Strongly disagree–10 = Strongly agree)	2.39 (2.19)
I sometimes feel God is punishing me for what I've done or not done while caring for patients. (1 = A great deal–10 = Not at all)	1.56 (1.56)

∗ Reverse coded before calculation according to instrument scoring instructions.

Note: Higher scores indicate greater MI.

### Impact of OE

**Figure 1:**
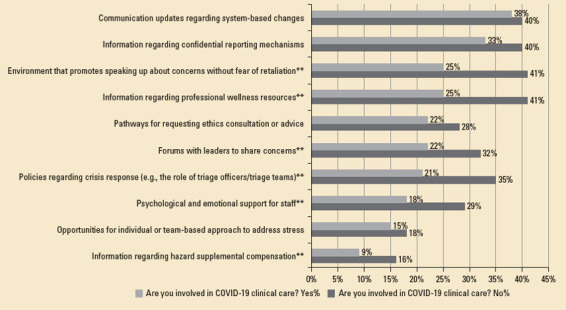
Relationship between providing COVID-19 clinical care and ratings of organizational effectiveness factors

Figure 1 displays the results of chi-square analyses examining the relationship between providing COVID-19 clinical care (0 = no, 1 = yes) and rating the facets of OE (0 = not at all/slightly/moderately effective, 1 = very/extremely effective). Nurses providing COVID-19 clinical care were significantly less likely to endorse “very/extremely effective” than nurses who didn't provide COVID-19 care on the following facets of OE: a) An environment that promotes speaking up about concerns without fear of retaliation (24.77% versus 40.94% respectively, *P* = .002); b) Information regarding professional wellness resources (24.54% versus 40.94% respectively, *P* = .0011); c) Forums with leaders to share concerns (22.12% versus 32.28% respectively, *P* = .038); d) Policies regarding crisis response (such as the role of triage officers/triage teams; 21.20% versus 34.65% respectively, *P* = -.006); e) Psychological and emotional support for staff (17.76% versus 29.13% respectively, *P* = .014); and f) Information regarding hazard supplemental compensation (8.80% versus 15.87% respectively, *P* = .047).

Table 3 summarizes the results of independent t-tests examining the relationships of the facets of OE (0 = not at all/slightly/moderately effective, 1 = very/extremely effective) coded with the total scores for MR and MI. Each facet of OE, apart from hazard/supplemental compensation, was significantly associated with both MR and MI. Nurses who responded that their organization was “very/extremely” effective with regards to each question had significantly higher mean MR scores and significantly lower mean MI scores compared with nurses responding that their organization was “not/slightly/moderately” effective.

**Table 3:** Organizational effectiveness item frequencies by moral resilience and moral injury scores, nurses only (N = 344)

		MR mean (SD)	*P* value	MI mean (SD)	*P* value
Communication updates regarding system-based changes	Not/slightly/moderately effective	28.25 (5.04)	.002	35.33 (12.24)	.002
	Very/extremely effective	30 (4.85)		30.26 (15.49)	
Information regarding confidential reporting mechanisms	Not/slightly/moderately effective	27.99 (4.97)	<.001	34.95 (13.4)	.004
	Very/extremely effective	30.69 (4.62)		30.2 (13.96)	
An environment that promotes speaking up about concerns without fear of retaliation	Not/slightly/moderately effective	28.15 (5.02)	<.001	35.76 (13.11)	<.001
	Very/extremely effective	30.8 (4.52)		27.66 (13.4)	
Information regarding professional wellness resources	Not/slightly/moderately effective	28.13 (4.87)	<.001	35.91 (13.06)	<.001
	Very/extremely effective	30.79 (4.91)		27.58 (13.74)	
Pathways for requesting ethics consultation or advice	Not/slightly/moderately effective	28.54 (5)	.009	34.99 (13.8)	<.001
	Very/extremely effective	30.2 (4.91)		28.35 (12.45)	
Forums with leaders to share concerns	Not/slightly/moderately effective	28.47 (5.04)	.003	35.35 (13.95)	<.001
	Very/extremely effective	30.26 (4.77)		27.24 (11.22)	
Policies regarding crisis response (such as the role of triage officers/triage teams)	Not/slightly/moderately effective	28.32 (4.94)	<.001	35.43 (13.68)	<.001
	Very/extremely effective	30.66 (4.87)		27.51 (12.33)	
Psychological and emotional support for staff	Not/slightly/moderately effective	28.41 (5.04)	<.001	35.58 (13.83)	<.001
	Very/extremely effective	30.85 (4.55)		25.59 (10.48)	
Opportunities for individual- or team-based approach to address stress	Not/slightly/moderately effective	28.65 (5.03)	.014	34.66 (13.68)	<.001
	Very/extremely effective	30.45 (4.78)		26.37 (12.13)	
Information regarding hazard supplemental compensation	Not/slightly/moderately effective	28.81 (4.95)	.238	33.92 (13.47)	.132
	Very/extremely effective	29.82 (5.52)		27.58 (13.74)	

### Predicting MI

As a preliminary step in building the regression model to predict MI, the bivariate comparisons, using analyses of variance or *t*-tests, of each predictor with MI were examined. Variables significantly related to MI at the bivariate level included: years in profession, religious/spiritual preference, MR score, OE score, and COVID-19 care. These significant predictors in preliminary bivariate analyses were included in the multivariate model. The following variables were significant predictors of MI and were added to the final model: OE score, MR score, 10-20 years working in the profession, and 20 or more years working in the profession. In addition, we examined the potential indirect impact of OE mediating the effect of COVID-19 care on MI. The final model (Table 4) explained 36% of the variability in MI score (*F* (6,522) = 27.72, *P* = .001, *R*^2^ = .36). All variables, except involvement in COVID-19 care and religious/spirituality, were significant predictors of MI (*P* < .05). After controlling for other variables in the model, for every one unit increase in OE, MI decreased by .40 points (*b* = -.40, *95%* CI *=* -.52, -.28), and for every one unit increase in MR, MI decreased by 1.14 points (*b* = -1.14, *95%* CI = -1.22, -.80). In addition, the indirect effect of providing COVID-19 care through OE on MI was statistically significant (coefficient = .74; *95%* CI = .04, 1.54) indicating that perception of OE mediated or was the mechanism through which providing COVID-19 care contributed to higher MI.

**Table 4:** Final regression model predicting moral injury (N = 307)

Factors	Adjusted b (95% CI)^a^	Standard error	*t*	*P* value^b^
MR score	-1.14 (-1.22,-.80)	.14	-8.30	<.0001
OE score	-.40 (-.52,-.28)	.06	-6.41	.0006
>20 years in profession	-5.27 (-8.24,-2.30)	1.15	-3.49	.0001
10-20 years in profession	-3.93 (-7.40,-.48)	1.76	-2.24	.03
COVID-19 care	.39 (-2.34,3.11)	1.39	.28	.78
Religion/spirituality	-1.66 (-5.69,2.38)	2.05	-.81	.42
Indirect/moderating effect of COVID-19 care and OE score	.74 (.04 to 1.54)	N/A	N/A	N/A

## Discussion

**Figure 2:**
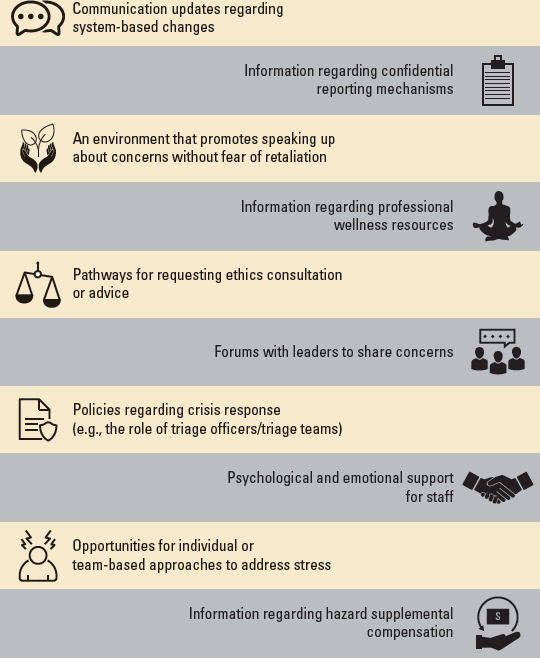
Factors of organizational effectiveness

Nearly 4 in 10 nurses from this sample reported clinically significant MI scores. MI symptoms have been associated with PTSD, depression, and suicidal thoughts.^13^,^15^ These are worrisome relationships that have been exacerbated during the COVID-19 pandemic and contribute to degraded personal well-being and work engagement.^16^,^17^ Organizational factors are thought to contribute to experiences of MI; therefore, this analysis aimed to understand OE from nurses' perspectives amid COVID-19 in relation to their MI and MR. We examined 10 different forms of OE (Figure 2), and other than hazard/supplemental compensation, each form significantly contributed to both MI and MR. Nurses who felt their organization was effective had higher MR and lower mean MI scores than nurses reporting their organization was less effective. Our analysis also demonstrates that higher OE and MR scores collectively contribute to MI scores. This suggests there may be areas within larger healthcare system structures that could contribute to morally injurious events but could also be modified to mitigate MI and bolster MR, concurrently. Our data suggest that investments to increase OE in these key areas are likely to decrease MI symptom scores and have the potential to reduce detrimental, longitudinal effects on nurses.

We found that MR was protective against symptoms of MI. This negative correlation between MR and MI is consistent with the overall group of interdisciplinary HCWs from the original study sample.^4^ A similar pattern was demonstrated in a study exploring the relationship of MR, moral distress, and mental health outcomes among HCWs.^7^ Increased MR scores were associated with lower moral distress, depression, stress, and anxiety.^7^ Our analysis demonstrated that higher OE and MR scores each uniquely contributed to MI scores. Thus, the combination of individual strategies to cultivate MR paired with system strategies that promote OE will have the highest potential for reducing symptoms of MI. This is contrary to claims by others that systemic change alone will stem the tide of MI.^18^,^19^

MR is an important protective resource when implementing interventions and/or system-level reforms, given its association with decreased burden and turnover intention.^20^ For example, programs such as the Mindful Ethical Practice and Resilience Academy (MEPRA) may have a positive impact on nurses and help protect against MI.^21^ MEPRA is an evidence-based program for nurses, designed to 1) enhance capacities associated with MR, and 2) cultivate resources such as mindfulness, ethical competence, and confidence, which allow nurses to respond to ethical challenges they face in their profession.^11^,^22^ Organizational investments in programs such as MEPRA have demonstrated increased work engagement and decreased turnover.^22^ When these programs are made available free of charge and with scheduling accommodations, nurses view this as evidence of their organizations' commitment to their employees' well-being, ethical competence, confidence, and ultimately, their MR. When organizations and leaders facilitate nurses' involvement in programs such as MEPRA, it helps rebuild trust and begins to heal the betrayals experienced throughout the pandemic.^9^

Increased length of time in the nursing profession, whether 10-20 years (-3.93 ± 1.76; *P* = .034) or greater than 20 years (-5.27 ± 1.15; *P* = .0001) was significantly associated with decreased MI; this was also observed in the overall sample.^4^ Growing evidence shows clinicians with more experience have lower levels of MI.^13^,^23^ These findings highlight the necessity of promoting protective factors to lower risk for MI in nurses in the early stages of their careers. This is critical given the higher proportion of this group who work in high-intensity settings, such as EDs and ICUs, and are often forced into leadership roles early in their career trajectory, all while coping with a mass exodus of more seasoned HCWs to positions in other locations or outside of healthcare completely.^23^,^24^ Accordingly, individual- and organization-level strategies as primary prevention must be implemented to reinforce MR for nurses at all stages, coupled with parallel efforts to identify and address the systemic root cause(s) of morally injurious events.^25^ Encouraging collaboration between clinical nurses, organization leaders, and other key personnel can enable trust in colleagues' capabilities and often lead to innovative solutions to address complex challenges.^9^,^11^,^26^

We found that nurses who provided COVID-19 care generally had poorer perceptions of OE than those who did not, which was consistent with a mixed-methods analysis from the overall study sample. Major themes were elicited from HCWs' written feedback, which highlighted the importance of several organizational factors that were absent or lacking.^9^ Of particular interest, psychological and emotional support for staff was rated effective by only 18% of nurses working with patients with COVID-19 versus 29% by those who weren't working with those patients. Similarly, information regarding professional wellness resources was rated effective by 25% of nurses working with patients with COVID-19, but by 41% of those not providing COVID-19 care. The contrast in how nurses providing COVID-19 care perceive OE could also be explained by the organizational stress associated with caring for patients with COVID-19 and its impact on nurses' appraisal of OE during crisis situations. Stress can reduce capacity to trust oneself and others when combined with chronic uncertainty; lack of resources; inconsistency in communication, decision-making, and policies; and broader societal unrest; therefore, it's likely to further erode nurses' perception of organizational trustworthiness.^26^ Organizational psychology posits that trust ultimately drives effective leadership, which has a significant trickle-down effect as it fosters a positive work environment, promotes HCW retention, and leads to improved patient-care delivery and outcomes.^27^-^29^

Unlike findings from our full sample of interdisciplinary HCWs, there were no statistically significant differences in MI comparing nurses who identified as spiritual or religious versus those who didn't. Previous work showed inverse relationships between religiosity and MI, suggesting spirituality may be protective against injury.^13^ Spiritual or existential conflict has been recognized as a theoretical core component of MI, with MI having the potential to erode an individual's sense of purpose and meaning in their work.^30^,^31^ These findings, that nurses may not rely on their spirituality or religious affiliation in the context of MI, may reflect that they've been existing in survival mode for the last 2+ years. This has left little room or energy for critical reflection or connection to their greater purpose.^4^

## Implications for nurse leaders

Our findings suggest opportunities for nurse leaders to design strategies to address the systemic factors that contribute to symptoms of MI. Clear and transparent communication regarding system changes, policies that impact crisis management, and the availability of wellness resources offer a starting point for rebuilding trust when it has been eroded.^9^,^26^ Given the chronicity of the pandemic, organizational leaders must engage with frontline nurses to better understand where they perceive gaps in process, content, and methods for delivering information at an institutional level.^32^ Examining organizational patterns that contribute to poor perceptions of mechanisms for confidential reporting of concerns, access to ethics consultation, individual and/or team wellness strategies, and stress management tools can help illuminate areas for intervention. Prioritizing relationships and investing in personal well-being is necessary to build back trust and enhance moral community and, as such, should be carefully prioritized in tailoring any interventions or programs aimed at improving MR among nurses.^9^,^32^,^33^ Amplifying and supporting the MR of nurses, combined with increased OE during crisis, offers a pathway to reduce the detrimental effects of unavoidable ethical challenges that contribute to MI while enhancing trust in healthcare leaders and organizations.

## Limitations

Results from this analysis should be contextualized within its limitations. We used a convenience sample rather than a random, representative sample. However, the sampling strategy did include nurses from a wide variety of institutions across several states. Although generalizations should be made with caution, the sample is reasonably representative. Second, we used a cross-sectional study design, making it difficult to determine causal relationships. Finally, all data were self-reported, so the relationships among various theoretical constructs may be inflated due to common method variance.

## A better system

Organizational decisions, polices, and resources influence nurses' perceptions of OE and contribute to the development of MI symptoms and MR. Nurse leaders are poised to address systemic factors that have contributed to the erosion of the nation's nursing workforce by further legitimizing the nursing leadership role and authority, empowering nurses to manage staffing, redesigning workflow, creating well-being boards to monitor indicators of nurse well-being, enhancing nursing autonomy, and engaging in interprofessional practice.^32^ From a sustainability perspective, HCWs, organization leaders, and researchers must collaboratively determine avenues for dismantling disempowering structures and patterns of betrayal within healthcare systems and bolstering MR for the betterment of nurses everywhere.

## Research criteria

**Purpose or goals:** To understand the relationship between nurses' perceptions of OE, MI, and MR during COVID-19

**Location and description:** Cross-sectional survey conducted in the mid-Atlantic US during the first wave of the COVID-19 pandemic; conducted secondary analysis using PROCESS macro for SPSS^®^ to carry out multiple regression analysis, including evaluation of indirect or mediator effect

**Population:** Subset of nurses from full original sample of interdisciplinary healthcare workers

**Collection tool(s):** Demographics, OE, MI, and MR

**Sample size:** N = 344

## INSTRUCTIONS Perceived organizational effectiveness, moral injury, and moral resilience among nurses during the COVID-19 pandemic: Secondary analysis

### TEST INSTRUCTIONS

- Read the article. The test for this nursing continuing professional development (NCPD) activity is to be taken online at www.NursingCenter.com/CE.
- You'll need to create an account (it's free!) and log in to access My Planner before taking online tests. Your planner will keep track of all your Lippincott Professional Development online NCPD activities for you.
- There's only one correct answer for each question. A passing score for this test is 7 correct answers. If you pass, you can print your certificate of earned contact hours and access the answer key. If you fail, you have the option of taking the test again at no additional cost.
- For questions, contact Lippincott Professional Development: 1-800-787-8985.
- Registration deadline is **June 6, 2025**.

### PROVIDER ACCREDITATION

Lippincott Professional Development will award 2.0 contact hours for this nursing continuing professional development activity.

Lippincott Professional Development is accredited as a provider of nursing continuing professional development by the American Nurses Credentialing Center's Commission on Accreditation.

This activity is also provider approved by the California Board of Registered Nursing, Provider Number CEP 11749 for 2.0 contact hours. Lippincott Professional Development is also an approved provider of continuing nursing education by the District of Columbia, Georgia, West Virginia, New Mexico, South Carolina, and Florida, CE Broker #50-1223. Your certificate is valid in all states.

Payment: The registration fee for this test is $21.95.

## References

[1] Buerhaus PI Staiger DO Auerbach DI Yates MC Donelan K. Nurse employment during the first fifteen months of the COVID-19 pandemic. *Health Aff (Millwood)*. 2022;40(1):79–85. 10.1377/hlthaff.2021.01289 34982625

[2] Ehli N. Short-staffed and COVID-battered, U.S. hospitals are hiring more foreign nurses. 2022. www.npr.org/sections/health-shots/2022/01/06/1069369625/short-staffed-and-covid-battered-u-s-hospitals-are-hiring-more-foreign-nurses.

[3] Mannino JE Watters P Cotter E The future capacity of the nursing workforce. *Nurse Educ*. 2021;46(6):342–48. 34261119 10.1097/NNE.0000000000001078 PMC8579884

[4] Rushton CH Thomas TA Antonsdottir IM Moral injury and moral resilience in health care workers during COVID-19 pandemic. *J Palliat Med*. 2022;25(5):712–719. 34678091 10.1089/jpm.2021.0076 PMC9081047

[5] Williamson V Murphy D Greenberg N. COVID-19 and experiences of moral injury in front-line key workers. *Occup Med (Lond)*. 2020;70(5):317–319. 32239155 10.1093/occmed/kqaa052 PMC7184422

[6] Azoulay E De Waele J Ferrer R Symptoms of burnout in intensive care unit specialists facing the COVID-19 outbreak. *Ann Intensive Care*. 2020;10(1):1–8. 32770449 10.1186/s13613-020-00722-3 PMC7414284

[7] Spilg EG Rushton CH Phillips JL The new frontline: exploring the links between moral distress, moral resilience and mental health in healthcare workers during the COVID-19 pandemic. *BMC Psychiatry*. 2022;22(19):1–12. 34991514 10.1186/s12888-021-03637-w PMC8734541

[8] Ulrich CM Rushton CH Grady C. Nurses confronting the coronavirus: challenges met and lessons learned to date. *Nurs Outlook*. 2020;68(6):838–844. 33097227 10.1016/j.outlook.2020.08.018 PMC7462465

[9] Nelson KE Hanson GC Boyce D Organizational impact on healthcare workers' moral injury during COVID-19: a mixed-methods analysis. *J Nurs Adm*. 2022;52(1):57–66. 34910709 10.1097/NNA.0000000000001103 PMC9199451

[10] Willard-Grace R Knox M Huang B Hammer H Kivlahan C Grumbach K. Burnout and health care workforce turnover. *Ann Fam Med*. 2019;17(1):36–41. 30670393 10.1370/afm.2338 PMC6342603

[11] Rushton CH. *Moral Resilience: Transforming Moral Suffering in Healthcare*. New York, NY: Oxford University Press; 2018.

[12] Heinze KE Hanson G Holtz H Swoboda SM Rushton CH. Measuring health care interprofessionals' moral resilience: validation of the Rushton Moral Resilience Scale. *J Palliat Med*. 2021;24(6):865–872. 33196347 10.1089/jpm.2020.0328

[13] Mantri S Song YK Lawson JM Berger EJ Koenig HG. Moral injury and burnout in health care professionals during the COVID-19 pandemic. *J Nerv Ment Dis*. 2021;209(10):720–726. 34582400 10.1097/NMD.0000000000001367

[14] Hayes AF. *Introduction to Mediation, Moderation, and Conditional Process Analysis: A Regression-Based Approach*. 2nd ed. New York, NY: The Guilford Press; 2018.

[15] Kelley ML Braitman AL White TD Ehlke SJ. Sex differences in mental health symptoms and substance use and their association with moral injury in veterans. *Psychol Trauma*. 2019;11(3):337–344. 30234322 10.1037/tra0000407 PMC6389381

[16] Lake ET Narva AM Holland S Hospital nurses' moral distress and mental health during COVID-19. *J Adv Nurs*. 2022;78(3):799–809. 34402538 10.1111/jan.15013 PMC8447301

[17] Saragih ID Tonapa SI Saragih IS Global prevalence of mental health problems among healthcare workers during the COVID-19 pandemic: a systematic review and meta-analysis. *Int J Nurs Stud*. 2021;121:104002. 34271460 10.1016/j.ijnurstu.2021.104002 PMC9701545

[18] Dyrbye LN Shanafelt TD Sinsky CA Burnout among health care professionals: a call to explore and address this underrecognized threat to safe, high-quality care. *Perspectives: Expert Voices in Health and Health Care*. 2017. https://nam.edu/wp-content/uploads/2017/07/Burnout-Among-Health-Care-Professionals-A-Call-to-Explore-and-Address-This-Underrecognized-Threat.pdf.

[19] West CP Dyrbye LN Shanafelt TD. Physician burnout: contributors, consequences and solutions. *J Intern Med*. 2018;283(6):516–529. 29505159 10.1111/joim.12752

[20] Antonsdottir I Rushton CH Nelson KE Heinze KE Swoboda SM Hanson GC. Burnout and moral resilience in interdisciplinary healthcare professionals. *J Clin Nurs*. 2022;31(1-2):196–208. 34145678 10.1111/jocn.15896

[21] Rushton CH Swoboda SM Reller N Mindful Ethical Practice and Resilience Academy: equipping nurses to address ethical challenges. *Am J Crit Care*. 2021;30(1):e1–e11. 33385208 10.4037/ajcc2021359

[22] Holtz H Heinze K Rushton CH. Interprofessionals' definitions of moral resilience. *J Clin Nurs*. 2018;27(3–4):e488–e494. 28771909 10.1111/jocn.13989

[23] Auerbach DI Buerhaus PI Staiger DO. Millennials almost twice as likely to be registered nurses as baby boomers were. *Health Aff (Millwood)*. 2017;36(10):1804–1807. 28971926 10.1377/hlthaff.2017.0386

[24] Yong E. Why health-care workers are quitting in droves. *The Atlantic*. 2021. www.theatlantic.com/health/archive/2021/11/the-mass-exodus-of-americas-health-care-workers/620713.

[25] Committee on Systems Approaches to Improve Patient Care by Supporting Clinician Well-Being. National Academies of Sciences, Engineering, and Medicine. *Taking Action Against Clinician Burnout: A Systems Approach to Professional Well-Being*. Washington, DC: The National Academies Press; 2019. 31940160

[26] Reina D Reina M. *Trust and Betrayal in the Workplace: Building Effective Relationships in your Organization*. 3rd ed. Oakland, CA: Berrett-Koehler Publishers; 2015.

[27] Dirks KT Ferrin DL. The role of trust in organizational settings. *Organ Sci*. 2001;12(4):450–467.

[28] Aquilia A Grimley K Jacobs B Nursing leadership during COVID-19: enhancing patient, family and workforce experience. *Patient Exp J*. 2020;7(2):136–143.

[29] Billings J Ching BCF Gkofa V Greene T Bloomfield M. Experiences of frontline healthcare workers and their views about support during COVID-19 and previous pandemics: a systematic review and qualitative meta-synthesis. *BMC Health Serv Res*. 2021;21(923):1–17. 34488733 10.1186/s12913-021-06917-z PMC8419805

[30] Jinkerson JD. Defining and assessing moral injury: a syndrome perspective. *Traumatology*. 2016;22(2):122–130.

[31] Litz BT Stein N Delaney E Moral injury and moral repair in war veterans: a preliminary model and intervention strategy. *Clin Psychol Rev*. 2009;29(8):695–706. 19683376 10.1016/j.cpr.2009.07.003

[32] Schlak AE Rosa WE Rushton CH Poghosyan L Root MC McHugh MD. An expanded institutional- and national-level blueprint to address nurse burnout and moral suffering amid the evolving pandemic. *Nurs Manage*. 2022;53(1):16–27. 10.1097/01.NUMA.0000805032.15402.b3 PMC8769498 34979524

[33] Nelson KE Rushton CH. Working while ill during COVID-19: ethics, guilt, and moral community. *AACN Adv Crit Care*. 2021;32(3):356–361. 34490445 10.4037/aacnacc2021342

